# Effects of Cultural Intelligence and Imposter Syndrome on School Belonging through Academic Resilience among University Students with Vocational Backgrounds

**DOI:** 10.3390/ijerph19137944

**Published:** 2022-06-28

**Authors:** Shiyong Wu, Wenxin Chen, Wei Chen, Wen Zheng

**Affiliations:** 1 South China Vocational Education Research Centre, South China Normal University, Foshan 528225, China; 2 School of Education, South China Normal University, Guangzhou 510631, China; 2021020688@m.scnu.edu.cn; 3 School of Education, Huizhou University, Huizhou 516000, China; zhengwen@hzu.edu.cn

**Keywords:** cultural intelligence, imposter syndrome, academic resilience, school belonging, transition, vocational pathway undergraduate

## Abstract

Background: University students with vocational qualifications encounter more severe cultural, academic, and self-evaluated challenges in the transitional process than their peers with an academic pathway. This study investigated the predictive effect of cultural intelligence (CI), imposter syndrome (IS), and academic resilience (AR) on school belonging (SB) and their interplay mechanism from a positive and negative perspective. Method: We recruited 326 Chinese university students with a vocational route as the research subjects and designed a parallel mediation model to assess the hypothesized construct. Result: The participants had scores above the median in CI, AR, and SB, but they also obtained scores exceeding the median in moderate IS. CI positively and significantly predicted SB both directly and indirectly through AR, while IS negatively and significantly predicted AR. AR both partly mediated the effect of CI on SB and entirely mediated the impact of IS on SB. Conclusion: CI was the most crucial factor impacting SB, followed by AR and IS among Chinese university students with a vocational education and training (VET) pathway. Strategic interventions should be adopted to enhance their abilities to cope with diverse cultures, promote their resilience in facing academic difficulties, boost their self-achievement, and foster their sense of SB.

## 1. Introduction

School belonging (SB) of university students has garnered considerable attention from policymakers, scholars, educators, and parents, along with the increasing diversity and openness of the higher education system worldwide in recent years [1,2,3,4]. Goodenow and Grady [5] conceptualized SB as “the extent to which they feel personally accepted, respected, included, and supported in the school social environment.” As an emotional and psychological construct, university students’ sense of SB reflects their positive attachment to the collegiate community and their culturally and academically transitional adaptation to a new surrounding [6,7,8]. Scholars have identified crucial predictors of SB in university settings that can be categorized into positive and negative facets [9,10]. Regarding the positive factors, the culturally relevant antecedents (e.g., cultural intelligence, CI) have been significantly linked to enhancing the sense of SB among the university population [11,12]. Moreover, the academically relevant determinants (e.g., academic resilience, AR) have also been associated with increasing university students’ SB [13,14]. Concerning the negative factors, students’ feelings of inadequacy or fraudulence (e.g., imposter syndrome, IS) manifesting as a sense that they successfully entered a university as a result of luck instead of their abilities have been related to decreasing their sense of SB [15].

CI refers to an individual’s capacity to perform effectively in culturally diverse settings [16,17]. CI is a widely accepted term that is well documented in academic research in the education system, linked to students’ emotional intelligence and intelligence quotient [18]. CI can help students adapt to unfamiliar contexts and blend in confidently [19]. Previous studies have shown that students with high CI are more effective at feeling supported by and connected to their institutions [20,21].

IS refers to individuals’ belief that their achievements are due to dumb luck or even error, persisting that they are not as intelligent or competent as others impress, despite abundant evidence to the contrary [22,23]. People suffering from IS are characterized by perfectionism, self-doubt, attributing their successes to external factors [24,25], and lack of confidence and belongingness [26,27]. The imposter phenomenon is also prevalent in higher education, especially for undergraduates and postgraduates [23,28]. Chapman [15] argued that students who can effectively overcome IS usually gain a great sense of SB by developing a new student identity.

AR refers to students’ ability to accomplish favorable learning outcomes despite a disadvantaged situation [29]. Some theoretical and empirical studies on predictors and outcomes of AR have confirmed that CI, IS, and SB are closely related to AR. According to the structural model of resilience [30], the factors affecting AR can be classified as protective and risk factors [31,32]. The protective factors fostering AR include intelligence and adaptation [33], while the risk factors weakening academic performance involve self-misrecognition of their worth [34]. For instance, extant research has revealed that the transition experience is a complexly cultural change that determines students’ academic performance [35] and is positively associated with AR for first-year students at higher education institutions [36]. In contrast, students who internalize themselves as inferior and inadequate with a high level of IS are prone to be less resilient when facing academic difficulties [37]. In addition, several studies have highlighted that AR is a positive predictor of SB [38,39]. Students with a high level of AR have a stronger sense of belonging to their universities [40], while those who have difficulties handling academic setbacks are less likely to form an appropriate sense of SB [41,42].

These existing studies have deepened our understanding of university students’ sense of SB and its antecedents in the transitional process. However, these representative determinants contributing to SB have not yet been integrated into a comprehensive model that can determine positive and negative factors. Specifically, little is known about the relationships between CI, IS, AR, and SB. Additionally, since SB is a growing domain at the collegiate level and is much more diverse and complex at the individual characteristic level, VET pathway university students may differ in the experience of SB compared to their peers with an academic route. Due to the significant distinctions in curriculums and examinations between education sectors [43], students upgrading to university from the VET sector are more likely to encounter the cultural, academic, and psychological shock, which catalyzes the feelings of self-worthless and isolation from the university community and further impacts their transition success [37,40]. Thereby, there is great significance in exploring the effects of CI, IS, and AR on SB among VET pathway university students.

With is in mind, following the suggestions that future research on SB should focus on the specifically marginalized populations using a quantitative approach [10], this study aimed to examine the predictive effects of CI, IS, and AR on SB among Chinese university students with a vocational pathway. This study is a further step to our previous work investigating the linear impact of bicultural identity integration, self-esteem, AR, and interaction anxiousness on SB of VET pathway university students in China [40]. In the present study, we synthesized the SB research and AR theory and designed a parallel mediation model to assess the mechanism of the interplay among variables observed. This effort will add value to the theoretical and empirical literature on SB constructs and the AR model by evaluating the effects of CI and IS on SB through AR in a sample of Chinese university students with a vocational background. The following hypotheses were assumed:

**Hypothesis** **1** **(H1).** *CI positively predicts SB*.

**Hypothesis** **2** **(H2).** *CI positively predicts AR*.

**Hypothesis** **3** **(H3).** *AR positively predicts SB*.

**Hypothesis** **4** **(H4).** *CI positively predicts SB through AR*.

**Hypothesis** **5** **(H5).** *IS negatively predicts SB*.

**Hypothesis** **6** **(H6).** *IS negatively predicts AR*.

**Hypothesis** **7** **(H7).** *IS negatively predicts SB through AR*.

**Figure 1:**
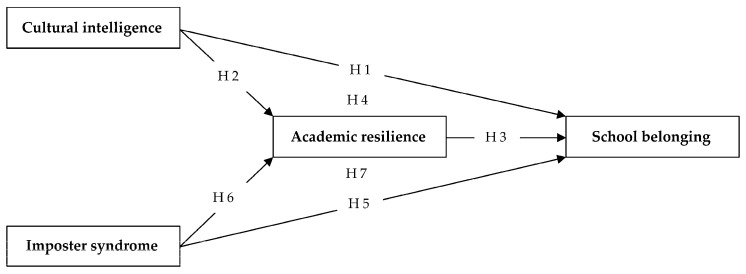
Posited model.

The hypothesized mediation model is presented in Figure 1.

## 2. Materials and Methods

### 2.1. Participants

The participants in this study were university students transitioning from the vocational education system and enrolling in a course taught by the first author to train students’ knowledge and skills in developing and filling in a questionnaire. The survey was distributed online using the Wenjuanxing survey software, also called Chinese Qualtrics, so that students could fill it out anonymously and conveniently. The data were collected from 5 to 12 January 2021; at that time, the participants had embedded into their university life for a half year. After the participants had delivered the survey, the data were filtered concerning whether the responses were random or inconsistent, such as long strings of invariant responses or extraordinary short response times. All potential participants responded to the survey; the valid number of responses was 326 after removing invalid answers. The research data were analyzed utilizing the bootstrapping procedure with 5000 subsamples to ensure the data representation.

### 2.2. Measures

#### 2.2.1. Cultural Intelligence Scale

CI was assessed with a nine-item Chinese version of the Cultural Intelligence Scales Short Form, initially designed by Ang and Dyne [12]. The responses were ranked on a seven-point Likert-type scale (1 = strongly disagree to 7 = strongly agree). The participants were asked to rate their levels of agreement with statements such as “I enjoy interacting with people from different cultural backgrounds.” Higher total scores mean higher CI. In the present study, the Cronbach’s alpha of this variable was 0.90.

#### 2.2.2. Imposter Syndrome Scale

IS was evaluated with a twelve-item Chinese version of the Imposter Syndrome Scale, initially developed by Clance [44]. This revised scale includes three dimensions: perfect (5 items), self-suspect (4 items), and external attribute (3 items) [45]. The responses were rated on a seven-point Likert-type scale (1 = strongly disagree to 7 = strongly agree). The participants were asked to rank their levels of agreement with statements such as “I am afraid that people who are important to me will find that I am not as capable as they think.” Higher total scores indicate higher IS. In this study, the Cronbach’s alphas for the total, perfect, self-suspect, and external attribute were 0.82, 0.66, 0.65, and 0.54, respectively.

#### 2.2.3. Academic Resilience Scale

AR was measured with a six-item Chinese version of the Academic Resilience Scale, originally conducted by Martin and Marsh [41]. The responses were ranked on a seven-point Likert-type scale (1 = strongly disagree to 7 = strongly agree). The participants were asked to rate their levels of agreement with statements such as “I think I am good at dealing with schoolwork pressures.” Higher total scores signify better AR. The Cronbach’s alpha of this variable was 0.92 in the current study.

#### 2.2.4. School Belonging Scale

SB was tested with a five-item Chinese version of the School Belonging Scale, developed by Zhu and Han [46], who extracted from the measures of Bollen and Hoyle [47] and Anderman [48]. The responses were marked on a seven-point Likert-type scale (1 = strongly disagree to 7 = strongly agree). The participants were asked to rank their degrees of agreement with statements such as “I am interested in what happened at school.” Higher total scores denote a stronger sense of belonging to the university. The Cronbach’s alpha of this variable was 0.90 in the present study.

### 2.3. Data Analysis

The data were analyzed with SPSS v.25, designed by the American International Business Machines Corporation (IBM) headquartered in Armonk, NY, USA, and SmartPLS v.3.3 software, developed by SmartPLS GmbH Company in Oststeinbek, Germany. First, the common method variance test was conducted using Harman’s single factor test [49] to ensure that the results were not biased by the instrument artifacts [50]. The result indicated that the single-factor solution explained only 28.99% of the variance, less than the recommended 40% threshold [51]. Hence, common method bias did not determine the results. Then, descriptive statistics involving means, standard deviations, and correlation analysis were conducted to assess the associations between variables observed. Next, given that this study mainly identifies which exogenous variables contribute to endogenous variables, rather than verifying a conceptual theory, the PLS-SEM method with measurement and structural model tests was used to assess the psychometric features of the posited model [52]. The measurement model was tested by the factor loading, internal consistency reliability, convergent validity, and discriminant validity, as suggested by Roldán and Sánchez-Franco [53]. The structural model was examined by the multicollinearity using the variance inflation factor, goodness of fit indices, and path coefficient beta value to test the hypotheses, as advised by Hair et al. [54]. Finally, the importance-performance-map analysis [55] was used to identify the most influential determinant predicting the outcome variable.

### 2.4. Ethics

The South China Normal University Academic Ethics Committee reviewed and authorized this study. The research ethics approval was provided to the participants with the explanatory statement and consent form.

## 3. Results

### 3.1. Characteristics of Participants

The participants consisted of 209 women (64.1%) and 117 men (35.9%). Their ages spanned from 17 to 29 years (*M* = 20.39, *SD* = 1.79). Nearly 60% of the respondents came from rural areas. Approximately 93% of the participants transitioned to higher education with a direct pathway from higher vocational colleges than the remaining with an indirect two-wave pathway from a secondary vocational school to a higher vocational college and, eventually, to university. The disciplines they majored in were Business English (29.8%), Electronic Commerce (36.5%), and Internet Engineering (33.7%). The details of the participants’ demographic characteristics in the current study are presented in Table 1.

### 3.2. Descriptive Statistics

Table 2 displays the means, standard deviations, skewness, kurtosis, and correlations between variables and sub-variables. The absolute skewness and kurtosis values are close to 2, indicating that the data were normally distributed [56,57]. Participants obtained the highest scores in SB, manifesting that they are connected to their universities. Similarly, students had slightly higher scores than the median in CI and AR, demonstrating that they had overcome learning challenges and blended into the new environment. Noteworthily, students also gained scores above the median in IS, illustrating that they might experience imposter feelings of intrinsically considering themselves unqualified, unintelligent, or unsuccessful, although they had transitioned to a well-known university from a low-reputation vocational school.

Regarding the correlations of the variables observed, CI positively and significantly related with AR (r = 0.46; *p* < 0.01) and SB (r = 0.58; *p* < 0.01). Similarly, AR positively and significantly associated with SB (r = 0.50; *p* < 0.01). As expected, IS was negatively significantly linked to AR (r = −0.15; *p* < 0.01) and SB (r = −0.11; *p* < 0.05), but the coefficient values were low than 0.3, representing an extremely statistically small correlations [58].

### 3.3. Measurement Model Test

The measurement model test represents the data reliability and validity using the following criteria: factor loading, Cronbach’s alpha, composite reliability, and average variance extracted [53,59]. According to Hair et al. [54], the threshold values for factor loading, Cronbach’s alpha, composite reliability, and average variance extracted should be higher than 0.70, 0.70, 0.70, and 0.50, respectively. However, MacCallum et al. [60,61] recommended that item over 0.60 value is acceptable for a factor model with a small sample size. Therefore, to improve the model’s satisfactory level and cover all dimensions, items with a factor loading over 0.7 were retained, except for two items of IS with values above 0.6. As shown in Table 3, the internal consistency reliabilities of latent variables ranged from 0.74 to 0.92 for Cronbach’s alpha and from 0.83 to 0.94 for composite reliabilities, and the values of average variance extracted also exceeded the 0.5 requirements. Therefore, all constructs were considered reliable.

Discriminant validity was also considered a critical signal for testing the differences between each construct of the research model. To assess discriminant validity, the heterotrait–monotrait (HTMT) ratio was used to calculate the mean of the correlations of all constructs. Referring to Henseler et al. [62], the HTMT value should be less than 0.85. The results in Table 4 indicated that each construct was distinct from other constructs, showing HTMT ratios among two constructs less than the suggested value.

### 3.4. Structural Model Test

The structural model was assessed by three parameters: variance inflation factor, goodness of fit, and path coefficient. According to Hair et al. [54], the cutoff value of the variance inflation factor should be less than 5. The results displayed in Table 5 indicated that the issue of multicollinearity did not occur in the exogenous variables. The criteria of the goodness of fit include four parts: standardized root-mean-square residual (SRMR, <0.1 advised by Kline [63]), d_ULS and d_G (>0.05 suggested by Dijkstra and Henseler [64]), normed fit index (NFI, >0.8 proposed by Daire et al. [65]), and R^2^ value (>0.3 recommended by Hair et al. [54]). The results presented in Table 6 reveal that the hypothesized model has a convincing explanatory and predictive power.

The path coefficient values were computed using bootstrapping method with 5000 subsamples, and its significance could be ascertained by the *t*-statistics value above 1.96 and *p*-value below 0.05 [54]. For convenience, we integrated these three indices into the mediation analysis to assess the research hypotheses.

### 3.5. Mediation Test

Table 7 shows the hypotheses test results. Since the total effects of CI (*β* = 0.54, *t* = 11.82, *p* = 0.000) and IS (*β* = −0.11, *t* = −2.72, *p* = 0.007) on SB were significant, the mediating role of AR was confirmed. In the first mediation model, CI positively and significantly affected students’ feelings of SB (*β* = 0.42, *t* = 8.54, *p* = 0.000). CI also had a positively significant impact on AR (*β* = 0.50, *t* = 9.02, *p* = 0.000). Therefore, Hypotheses 1 and 2 were fully supported. Meanwhile, AR was found to predict SB positively and significantly (*β* = 0.24, *t* = 5.59, *p* = 0.000). Therefore, Hypothesis 3 was supported. Based on these results, the partially mediating effect was confirmed that AR mediated the relationship between CI and SB (*β* = 0.12, *t* = 3.70, *p* = 0.000), explaining 22.70% of the total effect (ab/c). Therefore, Hypothesis 4 was supported.

In the second mediation model, IS was also negatively and significantly predictive of AR (*β* = −0.24, *t* = −4.74, *p* = 0.000), supporting Hypothesis 6. However, IS was found to affect students’ sense of SB negatively but not significantly (*β* = −0.05, *t* = −1.32, *p* = 0.186), failing to support Hypothesis 5. Based on these results, the fully mediating effect was confirmed, i.e., AR mediated the association between IS and SB (*β* = −0.06, *t* = −2.39, *p* = 0.016), explaining 100% of the total effect. Therefore, Hypothesis 7 was supported.

**Figure 2:**
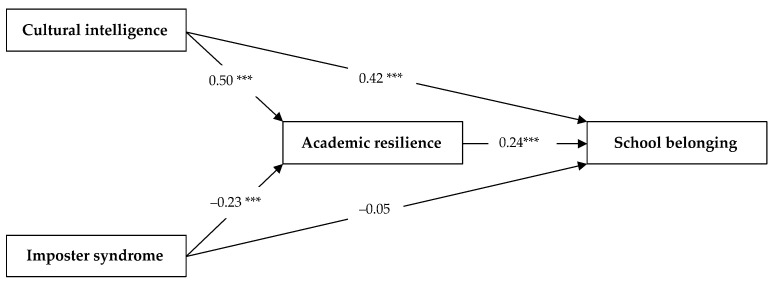
Mediation test. *** *p* < 0.001.

The complete pathway test results are presented in Figure 2.

### 3.6. Importance-Performance Map Analysis

Importance-performance analysis was conducted to identify the critical constructs by contrasting the total effects of the latent variables on a specific target variable with their average latent variable scores [54]. More precisely, the total effects of the constructs represent the importance of shaping the target constructs (in this study, referring to SB), while their average latent variable scores manifest their performance [66]. In this study, the target construct refers to SB, while the predecessor constructs include CI, IS, and AR. This useful analysis can extend the results representation of the PLS-SEM and has been widely utilized in education research [67,68]. Table 8 presents the results of the direct, indirect, and total effects (namely importance) of CI, IS, and AR on SB, as well as their mean scores (namely performance). Specifically, CI is the most important factor in predicting students’ SB (0.55) and obtained the highest mean score (69.52) compared with AR (0.28; 68) and IS (−0.11; 53.85).

**Figure 3:**
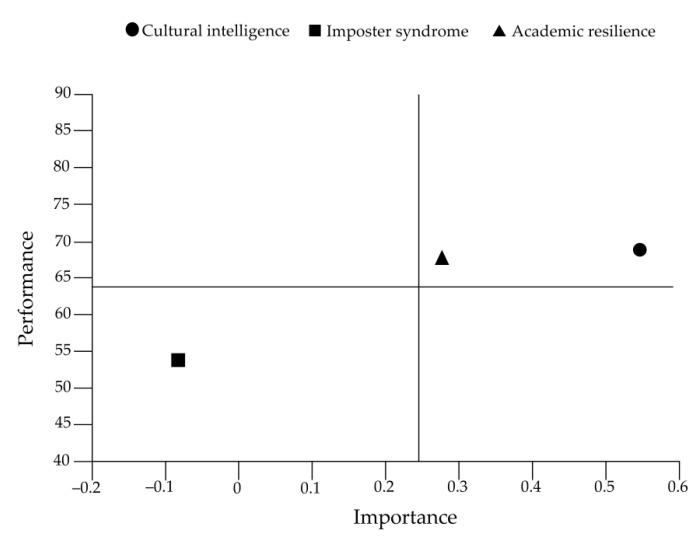
Importance-performance map.

To further position the potential areas of improvement that should pay great attention and action, an importance-performance map was created to visualize the results. The horizontal axis represents the importance, and the vertical axis represents the performance. According to Martilla and James [69], the importance-performance map could be divided into four quadrants: “keep up the good work” in the higher right, “concentrate here” in the lower right, “possible overkill” in the higher left, and “low priority” in the lower left. The variables in the lower right area are of the highest attention to accomplish improvement, followed by the higher right, the lower left, and the higher left areas [66]. Figure 3 depicts the positions of different variables in the importance-performance map. CI and AR are in high importance and performance areas, while IS resides in those with low importance and performance.

## 4. Discussion

This study sought to determine the effects of CI and IS on SB through AR among Chinese VET pathway university students. Participants’ overall means were over the midpoint of the scales, manifesting that they can make good use of intelligence to adapt to the cultural differences between university and vocational settings, effectively deal with difficulties and frustrations in professional learning, and feel supported and affirmed by the university. However, they also moderately struggled with the feeling of IS. This result coincides with previous studies wherein university students with vocational backgrounds generate self-misrecognition of their worth in higher education and their subject units relative to their peers with academic pathways [34]. This result may be due to the challenges of transitioning between different types of education proposed by Schlossberg [70] and Barber and Netherton [71]. Furthermore, structural equation modeling provided empirical support for our hypotheses of the intrinsic association between the participants’ CI, IS, AR, and SB. The importance-performance map analysis also identified the variables that had the most significant impact on SB and that should be shown more attention. The specific interrelationship between the four observed variables is elaborated in the following subsections.

### 4.1. The Relationship between CI, AR and SB

The results confirmed that CI had the most significant influence on SB among Chinese VET pathway university students. The first hypothesis test showed that CI positively and significantly affected students’ sense of SB. Thus, improving sensitivity and ability to cope with cultural affairs will promote students’ feelings of belonging to the university. This deduction is supported by Du’s finding [3] that vocational education students’ sense of SB can be cultivated from a cultural perspective. This result is also consistent with the conclusion by Ang and Dyne [12] that CI is strongly associated with SB. CI reflects an individual’s capability to cope with diversely cultural situations [16]. People who adapt to diverse cultural circumstances are more likely to shape a sense of belonging to new environments [12]. Therefore, the higher students’ CI was, the stronger their sense of SB was.

The second hypothesis was verified by the positive and significant effect of CI on AR. This result provides empirical evidence for previous theoretical arguments. According to the transition theory assumed by Schlossberg [72], when stepping into new learning surrounding, students are prone to encounter transition shock, including cultural shock [73] and learning shock [74]. When exposed to an unfamiliar and culturally diverse environment, individuals may feel stressed and frustrated in achieving life and academic success that requires psychological resilience to engagement [75]. Thus, CI, as a complementary type of intelligence that interprets adaptability to variety and cross-cultural communications [17], can facilitate an individual’s academic performance in tackling learning difficulties. This result is also akin to the empirical findings that demonstrated a positive correlation between CI and AR [76,77]. For instance, Jurásek et al. [78] found that the CI of university students indirectly affects AR through their adaptability. Grounded on these findings, it can be asserted that CI is a pivotal predictor of AR and should be considered when committing to investigating the inner AR levels among Chinese university students from vocational education.

The third hypothesis was also supported since the results disclosed that AR positively and significantly predicted SB, indicating that the higher students’ AR was, the stronger their feelings of SB became. This result aligns with the finding that AR was predictive of students’ SB [10,38]. As mentioned above, resilient students who regard learning differences and academic challenges as positive stimulus are more inclined to feel supported and have a sense of belonging to the institution [20,21]. Conversely, those who suffer difficulties in familiarizing themselves with university pedagogy and learning patterns struggle to achieve academic success and feel disconnected from their university community [42].

Therefore, the fourth hypothesis emerged from this research that CI had a positive and significant effect on SB through AR. This result further confirms the transition theory and SB research that students with high CI have better AR and stronger SB [40,79]. Noteworthily, both direct and indirect effects of CI on SB were significant, indicating that AR functioned as a partial mediator. Thus, there may be other contributors that were not considered in this study.

Moreover, the importance-performance map analysis results illustrated that CI and AR are the areas where improvement activities should be maintained to enhance their performance.

### 4.2. The Relationship between IS, AR and SB

In this study, IS was found to affect students’ SB, which was insignificant negatively. Therefore, Hypothesis 5 was not supported. This result contradicts the findings of Rackley [80], who advocated that impostorism negatively impacted students’ well-being and sense of belonging at college. This result is also evident from the descriptive analysis that IS had a weak link to SB. Based on their responses to the questionnaire, although students are somewhat self-doubting about their competencies and self-abasing in comparison with their academic pathway counterparties, they were thoroughly satisfied with and proud of their successful progression to a high-ranking university as a stereotyped vocational student [81] with a strong feeling of membership [40]. Therefore, IS did not show a significant influence on SB.

For Hypothesis 6, IS was found to negatively and significantly affect AR. According to the resilience theory, the risk factors such as pessimism and diffidence contribute to high cognitive impairment and low academic performance. The strength of IS can lead to the decline of AR [82]. Due to the considerable distinctions in pedagogy and teaching methods between vocational and academic education systems, university students with vocational backgrounds who internalized their inferiority were less flexible and buoyant in overcoming academic challenges. This result is similar to the findings of Thompson et al. [83] that undergraduates’ IS restricted the opportunity to achieve high academic performance and lowered their self-congratulation and self-esteem. This result is also likened to the findings of Walker [84], who demonstrated a significant negative relationship between the impostor phenomenon and academic self-efficacy, which was associated with, and a significant determinant of, AR [85]. Therefore, IS is a critical predictor of AR among Chinese university students shifting from vocational education.

Notably, despite the insignificant direct effect of IS on SB, the indirect impact of IS on SB through AR was significant, indicating that Hypothesis 7 was supported. In this study, AR completely mediated the association between IS and SB among vocational route university students. The overlapping of students’ high IS and low AR can prominently impede the growth of their sense of SB [86]. This result expands the previous literature on how IS influences students’ SB by highlighting the importance of AR in their transition process.

Moreover, according to the outcome of the importance-performance map analysis, IS is in the lower left area. As Ringle and Sarstedt [66], constructs in the third quadrant (below average importance and performance) are given the third priority. Thereby, although IS had the least importance and slightest effect on SB compared with CI and AR, given that it can negatively and significantly predict SB through AR, more improvement should be implemented to diminish students’ self-misrecognition to help them navigate the learning friction, and subsequently, to enhance their feelings of SB.

### 4.3. Implication

The results indicated that CI positively and significantly predicted SB both directly and indirectly through AR. The findings from the importance-performance map analysis also revealed that CI had the highest importance and performance in this posited model. Accordingly, keeping up the excellence of CI should be treated as the most essential work. In this regard, some measures should be considered to increase students’ sense of SB by accelerating their ability to perform effectively in diversely cultural contexts. Universities should provide more instruction and opportunities to help students adapt to the new community and develop their CI, including multidisciplinary curricular modules, cross-cultural school activities, and transitional experience sharing. Individually, students should engage in compulsory and optional cultural development events more proactively to build a close connection with the new environment and boost their sense of SB.

In addition, AR was also found to act as a mediator in the relationship between IS and SB. With its location in the first quadrant and higher prioritization in the importance-performance map, AR should also be viewed as a vital element. In this vein, universities and educators should offer more academic support to promote students’ academic performance and heighten their feelings of SB by reducing course load, improving teaching methods, facilitating academic training, and putting appropriate academic stress on their professional learning [87]. Individually, students should actively transfer the way of thinking and learning from practice-oriented to cognition-oriented, take an easy-to-difficult learning strategy [88], and bravely face the learning challenge to develop their AR.

Noteworthily, although IS only negatively and significantly affected SB through AR and was of the lowest priority among the three variables, IS was somewhat prevalent in this cohort of university students with vocational pathways. Therefore, more effective interventions focused on students’ beliefs about their ability should be initiated to lessen their self-underestimated syndrome to improve their AR and thus their sense of SB. Specifically, educators and parents should encourage students to recognize their worth, build confidence in their success, and regard transition based on their values and beliefs. University students with vocational qualifications should also be aware of the importance of transition experiences, understand themselves comprehensively and objectively in the changing environment, and adjust their mentality further to overcome the complex impact of two types of education.

## 5. Limitations and Further Work

Three limitations must be acknowledged in the current study. The first limitation is that despite the assurance of anonymity and response time, this research topic is a sensitive one with the data collected by self-reporting, which may raise the social desirability bias issue and restrict the causality of the findings. Therefore, further studies should adopt more ways to reduce this bias, such as designing bogus items or incorporating a simple self-reported item. The second limitation is that the participants came from only one well-known university, which restrains the generalizability of the findings. Therefore, future research should cover more respondents from different universities that recruit students from the vocational sector to make conclusions more representative and applicable. The third limitation is that we did not consider the differences in investigated constructs in controlled variables, such as gender, residence, pathway, and discipline. Further work could involve more demographical factors, such as family background and the student wellbeing/engagement program that the university provides, in the regression analyses to detail the distinctions between variables and improve the model validity.

## 6. Conclusions

By integrating the transition, SB, and AR theory, this research deepened our previous study to explore the mediating role of AR in the relationship between CI and SB and between IS and SB. The results revealed that VET route university students have relatively high levels of CI, AR, and SB. Meanwhile, they are also fraudulent and self-doubting when valuing their successful transition. The results also indicated that CI positively and significantly predicts AR and SB, while IS has a negatively and significantly predictive effect only on AR, but not SB. AR partly mediated the impact of CI on SB and completely mediated the effect of IS on SB. The importance-performance map analysis also identifies that CI is the most vital predictor of SB, followed by AR and IS. These empirical results expand our understanding of how positive and negative factors influence students’ mental health and well-being in transitioning from a vocational education sector to a university. This study also provides valuable insights into how to accelerate the VET pathway university students’ CI and AR, alleviate their feelings of IS, and ultimately reinforce their sense of SB by designing cross-cultural curricula and activities, developing their adaptability to new circumstances, facilitating their resilience in professional learning and academic training, enhancing their sense of self-fulfillment, and intervening early in the period of vocational education. This study could be further improved and generalized by broadening the sample diversity and including the control variables.

## Figures and Tables

**Table 1:** Characteristics of the study participants.

Characteristics	Sub-Characteristics	Number	Percentage
Gender	Man	117	35.9%
	Woman	209	64.1%
Age	17–20 years	213	65.3%
	21–25 years	108	33.1%
	26–29 years	5	1.6%
Residency	Rural	191	58.6%
	Urban	135	41.4%
Pathway	College to university	304	93.3%
	School to college to university	22	6.7%
Discipline	Business English	97	29.8%
	Electronic Commerce	119	36.5%
	Internet Engineering	110	33.7%

**Table 2:** Means, standard deviations, and correlations of the variables and sub-variables.

Variables	1	2	3	4	5	6	7	*M*	*SD*	Skewness	Kurtosis
1. CI	(0.90)							5.22	0.94	−0.76	1.83
2. IS	0.01	(0.82)						4.32	0.84	−0.44	1.02
3. Perfect	0.07	0.89 **	(0.66)					4.12	0.92	−0.41	0.32
4. Self-suspect	−0.01	0.87 **	−0.65 **	(0.65)				4.36	1.01	−0.41	0.84
5. External attribute	−0.06	0.77 **	0.52 **	0.53 **	(0.54)			4.58	1.05	−0.23	0.32
6. AR	0.46 **	−0.15 **	−0.06	−0.10	−0.26 **	(0.92)		5.08	1.12	−0.55	0.49
7. SB	0.58 **	−0.11 *	−0.08	−0.09	−0.12 *	0.50 **	(0.90)	6.02	0.95	−1.12	1.69

* *p* < 0.05, ** *p* < 0.01; *M*: mean, *SD*: standard deviation; CI: cultural intelligence, IS: imposter syndrome, AR: academic resilience, SB: school belonging; Cronbach’s alpha coefficients are presented in parentheses diagonally; The numbers 1, 2, 3, 4, 5, 6, and 7 in the first row represent the variables with the same numbers in the first column.

**Table 3:** Factor loading, Cronbach’s alpha, composite reliability, and average variance extracted of the variables.

Construct	Items	Factor Loading	Cronbach’s Alpha	Composite Reliability	Average Variance Extracted
CI	CI 2	0.74	0.90	0.92	0.60
	CI 3	0.78			
	CI 4	0.80			
	CI 5	0.77			
	CI 6	0.84			
	CI 7	0.73			
	CI 8	0.74			
	CI 9	0.71			
IS	Perfect 8	0.65	0.74	0.83	0.55
	Self-suspect 7	0.68			
	External attribute 10	0.79			
	External attribute 11	0.83			
AR	AR 1	0.76	0.92	0.94	0.73
	AR 2	0.83			
	AR 3	0.85			
	AR 4	0.91			
	AR 5	0.87			
	AR 6	0.86			
SB	SB 1	0.90	0.91	0.93	0.74
	SB 2	0.91			
	SB 3	0.90			
	SB 4	0.84			
	SB 5	0.71			

CI: cultural intelligence, IS: imposter syndrome, AR: academic resilience, SB: school belonging.

**Table 4:** Heterotrait–monotrait discrimination validity of the measurement model.

Variables	1	2	3	4
CI				
IS	0.18			
AR	0.51	0.34		
SB	0.62	0.24	0.54	

CI: cultural intelligence, IS: imposter syndrome, AR: academic resilience, SB: school belonging.

**Table 5:** Multicollinearity test results.

Exogenous Variables	Variance Inflation Factor	
	AR	SB
CI	1.02	1.29
AR		1.40
IS	1.02	1.11

CI, cultural intelligence; IS, imposter syndrome; AR, academic resilience; SB, school belonging.

**Table 6:** Fit indices of the structural model.

Fit Index	SRMR	d_ULS	d_G	NFI	R^2^
Proposed value	<0.10	>0.05	>0.05	>0.80	>0.30
Estimated value	0.07	1.21	0.46	0.83	0.39 (SB) 0.31 (AR)

SRMR: standardized root mean square residual, d_ULS: squared Euclidean distance, d_G: geodesic distance, NFI: normed fit index; SB: school belonging, AR: academic resilience.

**Table 7:** Result of the hypothesis test.

Hypotheses	Path	Coefficient	T	*p*	Decision
H1	cultural intelligence → school belonging	0.42	8.54	0.000	Supported
H2	cultural intelligence → academic resilience	0.50	9.02	0.000	Supported
H3	academic resilience → school belonging	0.24	5.59	0.000	Supported
H4	cultural intelligence → academic resilience → school belonging	0.12	3.70	0.000	Supported
H5	imposter syndrome → school belonging	−0.05	−1.32	0.186	Not supported
H6	imposter syndrome → academic resilience	−0.23	−4.74	0.000	Supported
H7	imposter syndrome → academic resilience → school belonging	−0.06	−2.39	0.016	Supported
Total	cultural intelligence → school belonging	0.54	11.82	0.000	Supported
Total	imposter syndrome → school belonging	−0.11	−2.72	0.007	Supported

**Table 8:** Direct, indirect, total effects/importance, and performance of the predecessor construct on SB.

Predecessor Construct	Direct Effect	Indirect Effect	Total Effect/Importance	Performance
CI	0.43	0.12	0.55	69.52
IS	−0.04	−0.07	−0.11	53.85
AR	0.28	–	0.28	68.00

CI, cultural intelligence; IS, imposter syndrome; AR, academic resilience.

## Data Availability

The data supporting this study’s findings are available from the corresponding author with the permission of South China Normal University upon request. Restrictions apply to the availability of these data, which were used under license for this study.
